# Childhood OSAS and Obesity: Prospective Associations of Anthropometric Markers With Objective Sleep Outcomes in the CHAT Trial

**DOI:** 10.1111/jsr.70156

**Published:** 2025-07-17

**Authors:** Catalina Ramírez‐Contreras, Verónica Palma Elgueta, Lautaro Briones‐Suárez

**Affiliations:** ^1^ Department of Nutrition and Public Health Faculty of Health and Food Sciences, University of Bío‐Bío Chillán Chile

**Keywords:** children, obesity, OSAS, prospective, sleep quality

## Abstract

Obstructive sleep apnea syndrome (OSAS) is characterised by episodic upper airway obstruction that occurs during sleep and is common in children with obesity. The aim of the present study was to analyse the association between baseline anthropometric markers and objective sleep quality measures at follow‐up in school‐aged children with OSAS. Four hundred and seven school‐aged children with OSAS (age 6.5 ± 1.4 years; 51.4% female, 92.1% non‐Hispanic or Latino) were included in this study from the Childhood Adenotonsillectomy Trial (CHAT) study database. For the analysis, general linear models and linear regression were tested for sleep quality outcomes using polysomnographic data at follow‐up (7 months) including: wake after sleep onset, sleep efficiency, sleep latency and total sleep duration and anthropometric markers at baseline including: waist circumference, neck circumference and obesity (body mass index percentile ≥ 95). Children with obesity had lower sleep efficiency (*p* = 0.021) and higher sleep latency (*p* = 0.049) than children without obesity. Additionally, higher neck circumference values were associated with lower total sleep duration (*p* = 0.042). At the same time, higher waist circumference was associated with lower sleep efficiency (*p* = 0.016) and lower total sleep duration (*p* = 0.013). Our study highlights the intricate relationship between childhood OSAS, obesity and neck and waist circumference that influences sleep outcomes. Our findings showed that obesity at baseline leads to poorer sleep quality, lower sleep efficiency and higher sleep latency at follow‐up, regardless of gender, age, ethnicity and adenotonsillectomy. These findings underscore the importance of addressing obesity, neck and waist circumference in paediatric OSAS.

**Trial Registration:** NCT00560859

## 1 Introduction

Obstructive sleep apnea syndrome (OSAS) is characterised by episodic obstruction of the upper airway that occurs during sleep and is common in children with obesity (Bhatt et al. 2021; Siriwat et al. 2020; Bachrach et al. 2022). In this context, the literature has shown that obesity is an important risk factor for the severity of OSAS in children (Siriwat et al. 2020; Caliendo et al. 2023), as excess adiposity increases the risk of OSAS by 24%–61% (Caliendo et al. 2023). The proposed mechanism involves the excessive accumulation of adipose tissue on the airway tissues, increasing pressure on the diaphragm, which, in turn, leads to deregulation of breathing, decreasing chest capacity and exerting pressure on the upper airway and muscles associated with the pharynx (Caliendo et al. 2023; Drager et al. 2013). Neck circumference is indeed a measure of excess adipose tissue on the upper body. Increases in neck circumference have previously been associated with the worst OSAS in children with obesity (Katz et al. 2021). On the other hand, waist circumference is used as a marker for central obesity. In fact, the risk of OSAS increases with the presence of abdominal obesity and increased waist circumference is associated with both the presence and severity of OSAS, especially in children with overweight or obesity (Bhatt et al. 2021). Obesity, especially central adiposity, contributes to airway obstruction through anatomical and functional mechanisms and is associated with increased systemic inflammation, insulin resistance and autonomic dysfunction in children with OSAS (Bhatt et al. 2021; Chuang et al. 2024; Liao et al. 2024). OSAS not only predisposes children to hypercapnia and hypoventilation, but also impairs later psychomotor development, as evidenced by deficits in basic learning functions such as memory and language (Mussi et al. 2023).

It is important to know that obesity affects sleep quality and starts a harmful cycle that impacts overall health (Ischander and Lloyd 2020; Elizabeth et al. 2021; Morrissey et al. 2020). People with obesity often have shorter sleep duration and poorer sleep quality, which can exacerbate weight gain (Chaput et al. 2016; Chaput and Dutil 2016; Li et al. 2017). A study in subjects with overweight or obesity found that sleep duration of less than 6 h was associated with increased body mass index (BMI) and poor sleep quality (Gupta et al. 2022). In a cross‐sectional study of adolescents in the United Kingdom, longer sleep latency was found to be independently associated with higher levels of adiposity and a greater likelihood of having overweight or obesity, particularly in females (Collings 2022). Sleep efficiency, on the other hand, was inversely related to adiposity indices. In a sample of Canadian children, sleep efficiency was inversely related to various adiposity indices (weight, waist, circumference, percentage of body fat, BMI *z* score and waist‐to‐height ratio), even after adjusting for variables such as physical activity and sedentary time (Mcneil et al. 2015). In another study, children with poor habitual sleep efficiency were reported to be 12 times more likely to have obesity than children with good habitual sleep efficiency (Elizabeth et al. 2021). Furthermore, in children with obesity, an environment without structured schedules for activities such as eating or sleeping has been observed to impair sleep efficiency (Elizabeth et al. 2021). Current evidence suggests a bidirectional relationship between circadian rhythm disruption and OSAS. Disorganised daily routines may lead to circadian desynchronization, which is associated with significant sleep disturbances and may exacerbate the pathophysiology of OSAS. In addition, OSAS contributes to altered circadian gene expression and dysregulation of biological rhythms through frequent awakenings and intermittent hypoxia, which may exacerbate associated metabolic, cardiovascular and neuropsychiatric comorbidities (Gabryelska et al. 2022; Šmon et al. 2023).

There is evidence that sleep deprivation can impair hormonal control over appetite and reduce physical activity due to the fatigue associated with sleep restriction, leading to weight gain (Elizabeth et al. 2021). Possible mechanisms linking obesity and insufficient sleep suggest that insufficient sleep is related to a hormonal imbalance that promotes feelings of hunger. Decreased leptin levels and increased ghrelin levels lead to increased appetite and calorie intake, which, in turn, contribute to obesity (Huang et al. 2024; Dashti et al. 2015). In this sense, paediatric OSAS is associated with significant disruptions in sleep architecture, including increased fragmentation, reduced REM sleep, lower sleep efficiency and shorter total sleep duration. These alterations, which tend to worsen with increasing OSAS severity, reflect a general decline in sleep quality compared to children without the condition (Don et al. 2024; Seo et al. 2021). Inadequate sleep duration, particularly, in children with concomitant obesity, has also been associated with unfavourable metabolic profiles, such as increased insulin resistance and abnormalities in glucose and triglyceride levels, increasing the risk of developing metabolic comorbidities (Bhushan et al. 2017).

The Controlled Randomised Childhood Adenotonsillectomy Trial (CHAT) is a multicenter, randomised, controlled trial comparing early adenotonsillectomy (eAT) with Watchful Waiting with Supportive Care (WWSC) in children aged 5–9 years with mild to moderate OSAS (Redline et al. 2011). Prior to the CHAT study, there were no definitive studies examining the effectiveness of supportive therapy not only in relieving sleep‐disordered breathing, but also in improving relevant outcomes such as quality of life. The study is important because it fills these gaps and provides evidence‐based data that can inform the clinical practice of paediatric OSAS treatment (Redline et al. 2011; Marcus et al. 2013).

Knowing the impact that obesity may have on OSAS and given the sparse evidence linking anthropometric measures to sleep quality in children with OSAS despite adenotonsillectomy (AT), the aim of the present study was to analyse the association between baseline anthropometric markers and objective sleep quality measures at follow‐up in school‐aged children with OSAS.

## 2 Methods

### 2.1 Participants and Study Design

A detailed description of this multicentre, single‐blind, randomised controlled trial of AT for OSAS in children has been published (Redline et al. 2011) and the primary cognitive and behavioural outcomes have been reported (Marcus et al. 2013). Briefly, children were eligible for study entry if they were 5–9.9 years of age, had a history of snoring, tonsillar hypertrophy and were considered to be surgical candidates for AT by an otolaryngologist. Exclusion criteria included a history of recurrent tonsillitis, extreme obesity (BMI *z* score ≥ 3), therapy for failure to thrive, medications for psychiatric or behavioural disorders (including attention deficit hyperactivity disorder), developmental delays requiring school accommodations and known genetic, craniofacial, neurologic or psychiatric conditions likely to affect the airway, cognition or behaviour. Children were screened further by standardised polysomnography (PSG). Children who had OSAS, defined as an obstructive apnea‐hypopnea index (AHI) between 2 and 30/h or an obstructive apnea index between 1 and 20/h and without prolonged oxygen desaturation time (arterial oxygen saturation [SpO2], 90% that was, 2% of total sleep time) were eligible for study participation. Children were randomised to either eAT (surgery within 4 weeks of randomisation) or to WWSC. Repeat PSG and anthropometry were performed at approximately 7 months after randomisation. The study was approved by the Institutional Review Board of each institution. Informed consent was obtained from caregivers and assent from children ≥ 7 years of age. The CHAT study was registered at Clinicaltrials.gov (NCT00560859).

The data use agreement for the current study was obtained by the first author (C.R‐C.) from the National Sleep Research Resource (Zhang et al. 2018).

### 2.2 Protocol

Anthropometric measurements were performed at baseline and at the 7‐month follow‐up using a standardised protocol by centrally trained and certified personnel. Measurements were performed by a two‐person team consisting of a ‘measurer’ and a ‘recorder’. Additionally, up to two nocturnal visits for PSG were performed (one before randomisation and another after 6 months), using techniques to ensure standardised data collection at all sites. All PSG data were processed, analysed and summarised at the Brigham and Women's Hospital Sleep Reading Center (SRC) using well‐developed quality assurance methods.

### 2.3 PSG

All children who participated in the study underwent PSG throughout the full night with study‐certified technicians using a standardised protocol according to the American Academy of Sleep Medicine (AASM) guidelines (Gregory et al. 2011). Evaluation was performed according to AASM paediatric criteria by certified technicians, who were blinded to all other study data, at two central PSG reading centres. The AHI was defined as the sum of all obstructive and mixed apneas and hypopneas associated with a 50% reduction in airflow and either desaturation greater than 3% or electroencephalographic arousal divided by total sleep time in hours. Sleep outcomes were defined as follows:

– Wake after sleep onset (WASO): the total duration between sleep onset and lights‐on/out‐bed time while being awake from Type I PSG. Values are expressed in minutes.
– Sleep efficiency: the ratio of sleep duration (i.e., total sleep time) to in‐bed period from Type I PSG. Values are expressed in percentage.
– Sleep latency: the interval between lights‐out/in‐bed time and sleep onset time from Type I PSG. Values are expressed in minutes.
– Total sleep duration: the interval between sleep onset and sleep offset while the participant is asleep, from PSG. Values are expressed in hours.

### 2.4 Anthropometric Markers

For the measurement, the children were instructed to wear loose, light clothing or a normal hospital gown. Weight (to 0.1 kg) was measured on a calibrated digital electronic scale. Standing height (to 0.1 cm) was measured using a calibrated wall‐mounted stadiometer with the child standing on stockings and weight was measured using a calibrated research‐grade digital scale. Neck and waist circumference (to 0.5 cm) were measured with a non‐stretchable fibreglass tape. The waist measurement was repeated three times, using the average values. The definition of obesity classification according to the US Centers for Disease Control and Prevention (CDC) was ≥ 95th percentile for age and sex (CDC 2024).

### 2.5 Statistical Analyses

Descriptive characteristics are presented for all participants, including mean and standard deviation for continuous variables and proportions for categorical variables. Two statistical models were used to respond to the research question: Are baseline obesity parameters predictive for child's sleep quality measures at follow‐up? (i) For continuous outcomes, general linear models were applied with baseline obesity (obesity BMI percentile ≥ 95) as the fixed factor and WASO, sleep efficiency, sleep latency and total sleep duration at follow‐up as the dependent variables. (ii) For continuous dependent and predictors, linear regression was applied with baseline neck and waist circumference as predictors and WASO, sleep efficiency, sleep latency and total sleep duration at follow‐up as the dependent variables. Models were adjusted for gender, age, ethnicity and arm to intervention (eAT) or control group (WWSC). All analyses were performed with the SPSS statistical computer software, version 29.0 (IBM SPSS Statistics, Armonk, NY, USA). Significance testing was considered when *p* < 0.05.

## 3 Results

A total of 407 schoolchildren (age 6.5 ± 1.4 years; 51.4% female, 92.1% non‐Hispanic or Latino) were included in this study (Table 1). Overall, the results showed that regarding anthropometric parameters at baseline, 33.2% of the sample has obesity. Regarding sleep quality measures at follow‐up, the mean value of WASO was 36.8 min, average sleep efficiency was 87.9%, sleep latency was 26.5 min and average total sleep duration was 7.7 h.

**TABLE 1:** Characteristics of the population studied.

Total sample (*n*)	407
Gender	
Females	209 (51.4%)
Males	198 (48.6%)
Age (years)	6.5 (1.4)
Ethnicity	
Hispanic or Latino	32 (7.9%)
Non‐Hispanic or Latino	375 (92.1%)
Intervention arms	
Early adenotonsillectomy (eAT)	199 (48.9%)
Watchful waiting (WWSC)	208 (51.1%)
Anthropometric markers at baseline	
Obesity^a^	135 (33.2%)
Waist circumference (cm)	62.3 (13.0)
Neck circumference (cm)	27.9 (2.9)
Sleep outcomes measures at follow‐up	
WASO (min)	36.8 (31.6)
Sleep efficiency (%)	87.9 (7.6)
Sleep latency (min)	26.5 (28.4)
Total sleep duration (h)	7.7 (0.8)

*Note*: Values are means (standard deviations) for continuous data and absolute values and percentages (%) for categorical data.

Abbreviation: WASO, wake up after sleep onset.

^a^ Obesity BMI percentile ≥ 95.

Results of comparison of sleep outcomes between children with OSAS with and without obesity at baseline are shown in Table 2. We observed that those children with obesity had lower sleep efficiency compared with those without obesity (*p* = 0.021). Specifically, children without obesity showed 1.927% higher sleep efficiency (95% CI: 0.297, 3.557) compared with children with obesity (Table 3). Regarding other sleep outcomes, we found that children with obesity had higher sleep latency than those without obesity. In detail, sleep latency was 6.138 min lower (95% CI: −12.239, −0.037) in children without obesity compared with children with obesity (Table 3).

**TABLE 2:** Comparison of sleep outcomes at follow‐up between children with OSAS with and without obesity at baseline (*n* = 407).

	Obesity at baseline		*p* ^a^
	Yes (*n* = 135)	No (*n* = 272)	
WASO (min)	40.27 (34.41)	35.15 (30.01)	0.225
Sleep efficiency (%)	86.60 (8.28)	88.58 (7.13)	**0.021**
Sleep latency (min)	30.13 (33.02)	24.78 (25.63)	**0.049**
Total sleep duration (h)	7.56 (1.00)	7.74 (0.77)	0.091

*Note*: Values are mean and standard deviation (SD) for continuous data. Significant *p*‐values are in bold.

^a^ Statistical analysis: general linear models were used to compare sleep outcomes at follow‐up between children with obesity and without obesity. Analyses were adjusted for gender, age, ethnicity and arm to intervention (eAT) or control group (WWSC).

**TABLE 3:** Differences of sleep outcomes at follow‐up between children with OSAS with and without obesity at baseline (*n* = 407).

	Without obesity at baseline	
	*β* [95% CI]	*p*
WASO (min)	−4.194 [−10.986; 2.597]	0.225
Sleep efficiency (%)	1.927 [0.297; 3.557]	**0.021**
Sleep latency (min)	−6.138 [−12.239; −0.037]	**0.049**
Total sleep duration (h)	0.158 [−0.025; 0.341]	0.091

*Note*: Data were analysed GLMs to calculate adjusted differences in sleep outcomes at follow‐up between children with OSAS with obesity and without obesity (reference group: with obesity). Analyses were adjusted for gender, age, ethnicity and arm to intervention (eAT) or control group (WWSC). The table shows the unstandardized coefficient (*β*), 95% CI and *p* value associated with each predictor variable. Significant *p*‐values are in bold.

As shown in Table 4, a significant association was found between total sleep duration and neck circumference. Specifically, we observed that higher values of neck circumference were associated with lower total sleep duration (*p* = 0.042).

**TABLE 4:** Associations between sleep outcomes at follow‐up and neck circumference at baseline in school‐aged children with OSAS (*n* = 406).

	Neck circumference (cm) at baseline	
	*β* [95% CI]	*p*
WASO (min)	0.787 [−0.554; 2.127]	0.249
Sleep efficiency (%)	−0.320 [−0.642; 0.002]	0.052
Sleep latency (min)	0.767 [−0.441; 1.975]	0.213
Total sleep duration (h)	−0.037 [−0.073; −0.001]	**0.042**

*Note*: Data were analysed using linear regression models to test associations between sleep outcomes at follow‐up and neck circumference. Analyses were adjusted for gender, age, ethnicity and arm to intervention (eAT) or control group (WWSC). The table shows the unstandardized coefficient (*β*), 95% CI and *p* value associated with each predictor variable. Significant *p*‐values are in bold.

Additionally, results of Table 5 show a significant association between sleep outcomes and waist circumference. Adjusted linear regression models showed that higher waist circumference was associated with lower sleep efficiency (*p* = 0.016) and with lower total sleep duration (*p* = 0.013).

**TABLE 5:** Associations between sleep outcomes at follow‐up and waist circumference at baseline in school‐aged children with OSAS (*n* = 406).

	Waist circumference (cm) at baseline	
	B [95% CI]	*p*
WASO (min)	0.230 [−0.065; 0.525]	0.125
Sleep efficiency (%)	−0.087 [−0.158; −0.016]	**0.016**
Sleep latency (min)	0.213 [−0.053; 0.479]	0.116
Total sleep duration (h)	−0.010 [−0.018; −0.002]	**0.013**

*Note*: Data were analysed using linear regression models to test associations between sleep outcomes at follow‐up and waist circumference. Analyses were adjusted for gender, age, ethnicity and arm to intervention (eAT) or control group (WWSC). The table shows the unstandardized coefficient (*β*), 95% CI and *p* value associated with each predictor variable. Significant *p*‐values are in bold.

## 4 Discussion

The main finding of this study was that children with OSAS and with obesity at baseline showed worst sleep outcomes at follow‐up. In particular, obesity in children was associated with lower sleep efficiency and higher sleep latency. Higher neck and waist circumference were associated with lower total sleep duration and only waist circumference was associated with lower sleep efficiency.

The results of a study in Spanish children showed that sleep latency decreased in line with BMI and fat mass before weight control was performed for 1 and 2 years of intervention (Catalán‐Lambán et al. 2023). Evidence suggests a close relationship between weight regulation in early life and establishing healthy sleep patterns. There is evidence of a close relationship between weight regulation at a young age and the establishment of healthy sleep patterns. On the other hand, a prospective cohort study reported that the most important variable associated with obesity in school‐aged children was habitual sleep efficiency (OR = 12.364 95% CI 1.352–113.028) (Elizabeth et al. 2021). The mechanism of how obesity exacerbates OSAS is by increasing the AHI, which is a measure of the severity of sleep apnea. The increased BMI in children with obesity is associated with a higher AHI, leading to more severe sleep‐disordered breathing and further reductions in sleep quality (Ischander and Lloyd 2020). In fact, the risk of OSAS is 1.74 times higher in children with obesity. This can be explained because obesity affects upper airway dynamics, as shown by increased airway collapsibility in children with obesity and OSAS, which contributes to intermittent hypoxia and further sleep fragmentation (Hasuneh et al. 2024). Obesity can lead to anatomical and functional changes in the upper airway, contributing to OSAS (Bachrach et al. 2022; Drager et al. 2013; Bitners et al. 2020; Dempsey et al. 2010). Indeed, a study using dynamic MRI has shown that adolescents with obesity and OSAS experience significant changes in airway cross‐sectional area during sleep, which are not observed during wakefulness. This suggests that sleep exacerbates airway collapsibility in children with obesity and OSAS (Bitners et al. 2020).

Consistent with our findings, other studies have shown that children with obesity and asthma experience poorer sleep efficiency and impaired gas exchange compared to their healthy‐weight counterparts. Also, obesity exacerbates these issues by prolonging light sleep stages (Conrad et al. 2022). Moreover, the interaction between obesity and OSAS can lead to a vicious cycle where poor sleep quality and reduced sleep duration contribute to further weight gain, which in turn worsens OSAS (Ischander and Lloyd 2020). Multidisciplinary weight loss interventions can improve OSAS severity and sleep duration, emphasising the role of obesity management in paediatric sleep health (Roche et al. 2020).

Remarkably, our results showed that children with a larger neck circumference had significantly lower total sleep duration. Excessive neck fat, as shown by a neck circumference above the 95th percentile for age and sex, can contribute to upper airway narrowing, a critical factor in the pathogenesis of OSAS (Katz et al. 2021, 2015). Children with larger waist and neck circumferences tended to have shorter sleep durations. The authors emphasised the importance of parental BMI and physical activity as factors that can mitigate the risk of sleep disturbances (Dave et al. 2022). Alternatively, a systematic review and meta‐analysis found that children with OSAS had a slightly greater waist circumference compared to controls, but the difference was not as pronounced as that for neck circumference (de Araújo Lopes et al. 2024). These results suggest that, although waist circumference is associated with obesity, neck circumference may be a stronger indicator of obstructive sleep apnoea and overall sleep duration. Therefore, increased neck circumference in children should be considered a clinical marker for the risk of sleep‐disordered breathing and thus also for a potential reduction in sleep duration due to OSA‐related sleep disorder.

Our study showed that waist circumference was the only significant predictor of low sleep efficiency. Consistent with our findings, a study of Canadian children aged 9–11 years (*n* = 515) found that sleep efficiency was negatively associated with waist circumference (Mcneil et al. 2015). It is important to note that the impact of waist circumference on sleep efficiency in children with OSAS is primarily mediated through the severity of sleep‐disordered breathing. Similarly to upper adiposity, the increased central adiposity, as indicated by a larger waist circumference, can exacerbate airway collapsibility and lead to more frequent apneic events. This leads to fragmented sleep and reduced sleep efficiency, as the child experiences frequent arousals and transitions between sleep stages (Mcneil et al. 2015; Dave et al. 2022). Therefore, routine measurement of waist circumference could be a useful measurement given that it is reproducible in the clinical setting and can be reliably performed by trained personnel, supporting its integration into routine assessment protocols for patients at risk of OSAS.

This study has several strengths, considering that it is a multicenter, single‐blinded, randomised, controlled trial with rigorous data collection that includes objective measures of anthropometric measures and sleep outcomes. In addition, the sample size is large and includes a diverse group of school children. However, several limitations should be noted when interpreting our results. First, the lack of a comparison group of children with obesity but without OSAS limits our ability to isolate the independent effects of OSAS. The inclusion of such a group would strengthen the interpretation of our results by clarifying whether the observed differences in sleep outcomes are primarily due to OSAS, obesity or their interaction. Future studies should consider including both OSAS and non‐OSAS comparison groups with and without obesity to more accurately determine the contributions of each condition. Secondly, it might be interesting to include in future studies different variables that could influence anthropometric and sleep variables in children with OSAS, such as environmental factors, parental habits or nutritional status, to name a few. Third, there is limited generalisability due to the inclusion and exclusion criteria of the CHAT study. Children with extreme obesity, developmental delays, genetic syndromes or neuropsychiatric conditions were excluded, which may limit the applicability of our findings to broader paediatric populations. Given the interplay between obesity and OSAS in children, lifestyle interventions such as structured exercise programmes, nutritional interventions and behavioural counselling can help reduce central obesity and improve sleep outcomes. Multidisciplinary strategies that combine weight management with sleep hygiene support may be particularly effective and should be investigated further.

## 5 Conclusions

In conclusion, our study emphasises the intricate relationship between childhood OSAS, obesity and neck and waist circumference, impacting sleep outcomes. Our findings showed that obesity at baseline leads to poorer sleep quality, lower sleep efficiency and higher sleep latency at follow‐up, regardless of gender, age, ethnicity and AT. Neck circumference emerged as a robust predictor of overall sleep duration, while waist circumference was more strongly associated with sleep efficiency. These results emphasise the importance of addressing obesity, neck and waist circumference to manage paediatric OSAS. Future research should investigate the underlying mechanisms linking these factors to sleep outcomes and explore targeted interventions to improve sleep quality in children with OSAS and obesity.

## Author Contributions

**Catalina Ramírez‐Contreras:** conceptualization, writing – original draft, formal analysis, methodology, writing – review and editing, data curation, visualization, investigation. **Verónica Palma Elgueta:** investigation, visualization, writing – original draft, methodology. **Lautaro Briones‐Suárez:** visualization, writing – original draft, investigation, methodology.

## Conflicts of Interest

The authors declare no conflicts of interest.

## Data Availability

The data that support the findings of this study are available from the National Sleep Research Resource. Restrictions apply to the availability of these data, which were used under license for this study. Data are available from https://sleepdata.org with the permission of National Sleep Research Resource.
